# Barriers and Factors Associated With Treatment Coverage of Severe Acute Malnutrition Among Children 6–59 Months in Agrarian and Pastoralist Areas of Ethiopia

**DOI:** 10.1111/mcn.70219

**Published:** 2026-07-01

**Authors:** Bayise Biru, Lieven Huybregts, Dessalegn Tamiru, Mohammed Areb, Alemayehu Haddis, Talla Fall, Mariama Touré, Tefera Belachew

**Affiliations:** ^1^ Department of Nutrition and Dietetics, Faculty of Public Health, Institute of Health Sciences Jimma University Jimma Oromia Ethiopia; ^2^ School of Public health, Institute of Health sciences Wallaga University Nekemte Oromia Ethiopia; ^3^ Nutrition, Diets, and Health Unit International Food Policy Research Institute (IFPRI) Washington District of Columbia USA; ^4^ College of Medicine and Health sciences Haramaya University Dire Dawa Ethiopia; ^5^ Department of Environmental science and Technology Jimma University Jimma Oromia Ethiopia; ^6^ Ethiopian Public Health Association Addis Ababa Ethiopia; ^7^ Nutrition, Diets, and Health Unit International Food Policy Research Institute (IFPRI) Dakar Senegal; ^8^ Ethiopian Midwives Association Addis Ababa Ethiopia

**Keywords:** children, Ethiopia, severe acute malnutrition, treatment coverage

## Abstract

Severe acute malnutrition remains a major public health concern, yet a large proportion of affected children do not receive appropriate treatment. Understanding the barriers and associated factors is essential for informing targeted, context‐specific interventions. A door‐to‐door screening campaign among children aged 6–59 months was conducted from May to September 2024 in Kersa (agrarian) and Jeldessa (pastoralist) woredas of Ethiopia. The treatment coverage of severe acute malnutrition was 12.8% (95% CI: 9.0%–17.9%). The most frequently reported barriers to treatment included lack of awareness of the child's nutritional status, time and financial constraints, and absence of family permission. Multivariable analysis revealed that household wealth index (aPR: 2.9; 95% CI: 1.5–5.5), use of water treatment methods (aPR: 4.6; 95% CI: 1.7–11.9), recent community MUAC screening (aPR: 12.0; 95% CI: 5.4–26.8), availability of treatment services at a nearby health post (aPR: 3.2; 95% CI: 1.1–9.9), and possession of a vaccination card (aPR: 3.2; 95% CI: 1.1–9.1) were significantly associated with severe acute malnutrition treatment coverage. The findings highlight that severe acute malnutrition treatment coverage in both pastoralist and agrarian settings of Ethiopia remains critically low. Stronger implementation of active screening and treatment services recommended by Ethiopian national guidelines, together with social protection measures for the poorest households, may improve treatment coverage.

**Trial Registration:** ClinicalTrials.gov (NCT06380504).

## Summary

- SAM treatment coverage was very low (12.8%), far below the sphere standard for rural settings.
- Lack of caregiver awareness of child nutritional status was the main barrier to seeking SAM treatment.
- Community‐based MUAC screening strongly increased the likelihood of children receiving SAM treatment.
- Availability of SAM treatment services at nearby health posts significantly improved treatment coverage.
- Children from wealthier households and households engaged in preventive health practices were more likely to access treatment.
- Only a small proportion of children had been screened for wasting in the community, indicating major gaps in active case‐finding.

## 1 Introduction

Severe acute malnutrition (SAM) remains one of the serious public health challenges affecting children under 5 years of age. In 2024, an estimated 42.8 million children under the age of five were affected by wasting at any given time, including 12.2 million with SAM, the majority of whom resided in Asia and Africa (UNICEF/WHO/World Bank Group 2025). In Ethiopia, acute malnutrition continues to pose a significant concern, with 11% of children under five affected as of 2023 (EPHI 2023).

SAM is a critical global health concern due to its profound short‐ and long‐term consequences (Gizaw et al. 2023; Sania et al. 2019). Undernutrition compromises the immune system, rendering children more vulnerable to infections and significantly elevating their risk of mortality (Rytter et al. 2014). Globally, undernutrition is estimated to contribute to nearly 50% of all child deaths (WHO 2024). Children suffering from SAM are 11.6 times more likely to die than their well‐nourished counterparts, underscoring the urgency of timely identification and treatment (Olofin et al. 2013).

Early identification and treatment of children affected by SAM are essential to achieve the targets set by the Global Action Plan on Child Wasting and to preventing the life‐threatening consequences of SAM (FAO, UNHCR, UNICEF, WFP, & W 2020) (United Nations Systems Standing Committee on Nutrition [UNSCN] 2017)(WHO 2023a). Despite the implementation of community management of acute malnutrition (CMAM) programs, many children with SAM are still diagnosed too late, which significantly increases their mortality risk (UNICEF 2021). Previous studies have shown that if the CMAM is well implemented, a large proportion of children with SAM can recover (Baye et al. 2025). However, programmatic data indicate that only a limited number of children suffering from SAM receive treatment. For instance, in 2019, only one in three children with SAM accessed treatment, highlighting a critical gap in early intervention and service coverage (FAO, UNHCR, UNICEF, WFP, & W 2020).

Multiple factors may contribute to low treatment coverage of SAM. Previous studies have identified barriers such as limited community awareness, misconceptions about wasting, stigma, caregiver workload, transportation challenges, and limited decision‐making autonomy among women. These factors can delay or prevent caregivers from seeking treatment for affected children. (J. R. Bliss et al. 2016; Puett and Guerrero 2015), (Ahmed et al. 2022; Alelign et al. 2024; Burtscher and Burza 2015).

Although Ethiopia implements a unified national CMAM program (FMOH, G. of E 2019), delivery challenges may differ by livelihood context. Comparing agrarian and pastoralist settings is conceptually important because these differences can affect both supply‐ and demand‐side access to SAM treatment. Understanding these context‐specific barriers can help optimize SAM treatment coverage across diverse populations.

As part of the baseline survey for the “Research to Improve Screening for Wasting and Identification and Treatment of Wasted Children (R‐SWITCH)” project, this study aimed to determine the treatment coverage of SAM and to identify its key determinants and perceived barriers in Ethiopia's agrarian and pastoralist regions.

## 2 Methods

### 2.1 Study Setting

This study was based on cross‐sectional baseline data from the large cluster randomized controlled trial, the R‐SWITCH project conducted in Ethiopia. Data collection was carried out in two purposively selected woredas: Kersa woreda of Jimma Zone, Oromia Region, which is predominantly agrarian, and Jeldessa woreda in Dire Dawa City Administration, which is predominantly pastoralist. Kersa woreda is located in the Jimma Zone of southwestern Ethiopia at a distance of 357 km from the capital, Addis Ababa. The woreda has 35 kebeles (the smallest administrative unit), with each kebele having its own health post staffed by two health extension workers (HEWs). Kersa woreda is predominantly rural, with a total population of 246,896 people, including 40,567 children under the age of five. In contrast, Jeldessa woreda has a smaller population of 32,590 people, with 3956 of them being under the age of 5 years. These two woredas were strategically selected for the study based on factors such as SAM prevalence, accessibility, diversity in livelihoods, and overall security status. The study involved 40 kebeles in these woredas, 32 from Kersa and 8 from Jeldessa.

In Ethiopia, Alliance for Development (AFD) groups, formerly known as the Women Development Army (WDA), are community‐based networks organized into one‐to‐five or one‐to‐thirty groups of volunteers who support the country's health and development goals. Their primary role is to work with HEWs to extend the reach of health services and encourage healthy practices at the household level.

### 2.2 Study Population

The study population consisted of children aged 6–59 months with SAM at the time of the survey or those currently enrolled in the outpatient therapeutic program (OTP) for SAM. These children were identified through a door‐to‐door screening campaign in which child anthropometry was measured among all children aged 6–59 months. Children with congenital malformations were excluded from the study due to the difficulty of obtaining accurate anthropometric measurements.

For the barrier assessment, we included caregivers of children with SAM, as well as a random sample of HEWs from health posts within the kebeles and AFD leaders. One HEW was randomly selected per health post. In kebeles with two health posts, two HEWs were interviewed; this approach was applied across all 40 study kebeles. Additionally, AFD leaders were randomly selected from each got (the smallest administrative unit within each kebele).

### 2.3 Study Outcomes

For this study, SAM was defined as weight‐for‐length/height Z‐score < −3 (relative to the WHO 2006 standard), and/or MUAC < 115 mm, and/or the presence of bilateral pitting edema (WHO 2023b).

SAM treatment coverage was defined as the proportion of children with SAM or recovering SAM enrolled in SAM OTP at the time of the survey. We calculated period coverage to provide a comprehensive estimate of the program's ability to recruit and retain cases.

Period treatment coverage of SAM was defined as the proportion of both active SAM cases (*C*_in_) and recovering cases (*R*_in_) currently enrolled in the treatment program, divided by the total number of SAM cases (*C*_in_ + *C*_out_) plus recovering cases (*R*_in_) identified during the survey. This estimator was chosen because it accounts for the successful retention of children from admission through the recovery phase, which is a critical indicator of program adherence.

SAM treatment period coverage =

$$\frac{C_{\text{in}}+R_{\text{in}}}{C_{\text{out}}+C_{\text{in}}+R_{\text{in}}}\times 100$$

A child was considered to be under treatment by the study protocol when a caregiver acknowledged that the child was enrolled in a treatment program, and the child was fed RUTF over the last 3 days and the caregiver can either show at least one full RUTF sachet or more than one empty RUTF sachet.

### 2.4 Sample Size Determination

The sample size was based on a preliminary calculation conducted for the cluster randomized controlled trial. A total of 40 clusters were required to detect a 15 percentage point difference in treatment coverage, assuming a design effect of 2, a cluster size of 17, a type I error of 5%, and a statistical power of 90%.

### 2.5 Data Collection Tools, Procedures and Measurements

Child weight was measured using a calibrated digital weighing scale (SECA, Germany) to the nearest 0.1 kg. Height was measured using a stadiometer in a standing position, with the head aligned to the Frankfurt plane (i.e., line of sight perpendicular to the vertical board), knees fully extended, heels together, and the shoulders, buttocks, and heels in contact with the vertical board. Measurements were recorded to the nearest 0.1 cm. For children under 2 years, recumbent length was measured to the nearest 1 mm using a length board. Mid‐upper arm circumference (MUAC) was measured on the bare left arm, midway between the olecranon and acromion processes, using a non‐stretchable measuring tape. All anthropometric measurements were taken in duplicate. If the first two readings differed beyond the acceptable range (300 g for weight, 5 mm for length and MUAC), a third measurement was taken, and the average of the values was used. Edema was assessed by applying gentle pressure to the dorsum of both feet for approximately 3 s. Prior to data collection, three sessions of standardization exercises were conducted involving repeated measurements on 10 children aged 6–23 months and 10 children aged 24–59 months. These exercises assessed both inter‐ and intra‐measurer variability, and additional training was provided until all data collectors met the required accuracy and precision benchmarks. For precision, a round of measurements was considered successful if the sum of squared differences of the measurer's repeated measurements was lower than two times the sum of squared differences of the repeated measurements by the expert/reference measurer. For accuracy, a round of measurements was considered successful if the sum of squared differences between the measurer and the expert/reference measurer was lower than three times the sum of squared differences of the expert's repeated measurements (Table S1).

Questionnaires were administered and data captured through computer‐assisted interviewing implemented in Survey Solutions (World Bank, Washington DC, USA). Questionnaires were prepared in English first, then translated into local languages (Afan Oromo and Amharic) and back translated by speakers of both languages. Data were collected on household composition, food insecurity, asset ownership (to generate a socioeconomic proxy score), and water, sanitation, and hygiene (WASH) conditions. At the caregiver level, information was gathered on socio‐demographic characteristics, maternal knowledge, decision‐making autonomy, symptoms of depression, and perceived stigma. The child's age was estimated using the date of birth shown on the vaccination card or birth certificate. If these were not available, the date of birth was estimated using a local events calendar using widely recognized holidays, religious events and agricultural milestones as reference points.

Perceived barriers to treatment‐seeking for SAM were assessed using an open‐ended question. The data were intended to provide descriptive information complementary to the analysis of determinants. The caregiver data were considered the primary source, while data from AFD leaders and HEWs were used to assess convergence with caregiver responses.

### 2.6 Data Management and Analysis

Data cleaning and statistical analyses were performed using Stata version 17 (StataCorp LLC, Texas, USA). To construct a household wealth index, principal component analysis (PCA) was conducted using data on household asset ownership (Vyas and Kumaranayake 2006). The wealth index tertiles were derived from the pooled samples from the two woredas. The indicators included possession of durable goods (radio, watch, mattress, plough, chair and solar panel), housing conditions (number of sleeping rooms), livestock ownership (sheep, goats, camels, bulls, cows, donkeys, mules, and chickens), possession of a bank account, being a member of community based health insurance (CBHI) and ownership of agricultural land. Variables with prevalence below 5% or above 95% were excluded from the analysis. The first principal component, which explained 22.4% of the total variance, was used as a proxy for household wealth and was categorized into tertiles for subsequent analysis. Detailed factor loadings and the proportion of variance explained by the model are provided in the supplementary file (Table S2).

Descriptive statistics were computed as mean ± standard deviation (SD) for continuous variables and as frequencies with percentages for categorical variables. Cluster‐robust standard errors (linearized variance estimator) were employed to calculate 95% confidence intervals of the treatment coverage. This approach is used to adjust for the potential correlation of outcomes among children within the same cluster (Kebele), ensuring that the precision of the coverage estimates is not overestimated. Mixed‐effects Poisson regression with robust variance estimation and adjusted for clustering by kebele (random effect) was used to regress treatment coverage over candidate determinants. We used a Poisson regression with robust variance estimation to directly estimate prevalence ratios (PR) and their 95% confidence intervals (95% CI). Robust standard error estimation was used to relax the assumption of a Poisson distribution for binary data.

Following the purposeful selection of covariates approach, variables with a *p*‐value less than 0.25 in the bivariable analysis were considered candidates for inclusion in the multivariable model to ensure that important confounders were not overlooked (David and Stanley Lemeshow 2013). In both models, kebele was treated as a random effect and woreda as a fixed effect to account for the hierarchical data structure.

Multi‐collinearity was assessed for all independent variables prior to the multivariable analysis. The results showed that all variables had a variance inflation factor (VIF) of less than 2.0, with a maximum VIF of 1.89 and a mean VIF of 1.24. These values are below the threshold of 5, indicating that there was no significant multi‐collinearity among the predictors. A backward stepwise elimination approach was applied to retain variables with a *p*‐value less than 0.1 in the final model. Initially, all the variables were included in the regression model. Then, the least statistically variables were sequentially removed, one at a time, until only those with a *p*‐value less than 0.1 remained in the final model. A conservative threshold of *p* < 0.1 was used for retention in the final model to minimize the risk of excluding important confounders and to ensure robust adjustment of the estimates. Statistical significance was set at *p* < 0.05. This distinction allowed us to maintain a stable model while adhering to a rigorous significance criterion for our conclusions.

### 2.7 Ethical Considerations

Prior to starting the study, ethical clearance was obtained from the Ethiopian Midwives Association Institutional Review Board (Reference No: EMwA‐IRB/0010/2024) and the Institutional Review Board from the International Food Policy Research Institute (ref no: NDH‐24‐0308). All members of the data collection and study team received training on the approved protocol and were bound by core ethical principles, including respect for persons, beneficence, and justice. Consent forms were translated into the local language to ensure comprehension. The study's purpose and procedures were explained to mothers/caregivers, heads of households of eligible children, HEWs, and AFD representatives using an information sheet in the local language. Consent for children was provided by a parent or legal guardian. The informed consent form was signed by the child's primary caregiver and, when present, by the head of household. Verbal consent was obtained from caregivers for the anthropometric measurements (screening), while written informed consent was obtained for the interview. For caregivers with low literacy, the study procedures and consent information were explained verbally in the local language, and they were allowed to give consent using a finger sign/thumbprint. All collected data were treated with strict confidentiality and were not disclosed in any form that could reveal personally identifiable information. Measures were taken to ensure anonymity throughout data collection, management, analysis, and dissemination. Completed questionnaires were automatically deleted from the interviewer's tablet following synchronization with a secure server. Children identified with SAM who were not already receiving treatment were referred to the nearest health facility to be enrolled in the existing OTP which is free of charge. The protocol of the study was prospectively registered on clinicaltrials.gov with ID NCT06380504.

## 3 Results

### 3.1 Characteristics of Children, Households, and Health System Factors

Out of a total of 27,962 children screened, 687 (2.4%) were identified as having SAM, with a prevalence of 2.3% in Kersa and 3.4% in Jeldessa. The mean age of children was 19.1 ± 13.0 months. Of the total participants, 302 (44.0%) were male. Overall, 43.0% of the children were severely stunted, and 43.2% were severely underweight. At the community level, 3.2% and 4.8% of children had undergone MUAC screening within 1 month and 3 months prior to the survey, respectively. An estimated 65.0% of households reported experiencing food insecurity. While 67.5% had access to an improved water source, only 7.1% utilized water treatment methods (Table 1).

**Table 1:** Characteristics of children aged 6–59 months with SAM, and their caregivers, households, and health posts in agrarian and pastoralist areas of Ethiopia, 2024 (*n* = 687).

Variables	Agrarian (*n* = 608)	Pastoralist (*n* = 79)	Total (*n* = 687)
Age of child in months	18.8 ± 12.5	21.1 ± 15.9	19.1 ± 13.0
Male	262 (43.1)	40 (50.6)	302 (44.0)
Vaccination card available	100 (16.5)	24 (30.4)	124 (18.1)
Length/height‐for‐age Z‐score	−2.8 ± 1.4	−2.1 ± 1.6	−2.7 ± 1.4
Length/height‐for‐age Z‐score < −2	441 (74.1)	40 (50.6)	481 (71.4)
Length/height‐for‐age Z‐score < −3	268 (45.0)	22 (27.8)	290 (43.0)
Weight‐for‐age Z‐score	−3.1 ± 1.0	−3.2 ± 1.1	−3.1 ± 1
Weight‐for‐age Z‐score < −3	271 (44.6)	26 (32.9)	297 (43.2)
SAM concordance^a^	62 (10.2)	10 (12.7)	72 (10.5)
SAM concordance^b^	1 (0.17)	0 (0.0)	1 (0.15)
WaST	422 (69.6)	39 (49.0)	461 (67.3)
**Child MUAC screened in the community** c			
In the last one month	16 (3.1)	3 (4.5)	19 (3.2)
In the last three months	24 (4.6)	4 (6.0)	28 (4.8)
Number of under five children	1.7 ± 0.7	1.9 ± 0.7	1.8 ± 0.7
**Household wealth index**			
Low	159 (26.2)	70 (88.6)	229 (33.4)
Medium	221 (36.4)	8 (10.1)	229 (33.4)
High	227 (37.4)	1 (1.3)	228 (33.2)
Household food insecurity	388 (64.0)	57 (72.2)	445 (65.0)
HFIAS Score	6.7 ± 6.5	8.3 ± 6.4	6.9 ± 6.5
Uses improved water source	430 (70.8)	33 (41.8)	463 (67.5)
Used water treatment methods	29 (4.8)	20 (25.3)	49 (7.1)
Has hand washing station with soap	198 (32.6)	3 (3.8)	201 (29.3)
Health post offers SAM OTP and has RUTF available^d^	149 (24.5)	48 (60.8)	197 (28.7)

*Note:* Data are presented as mean ± standard deviation (SD) for continuous variables and *n* (%) for categorical variables.

Abbreviations: HFIAS: Household Food Insecurity Access Scale; OTP: Outpatient Therapeutic Program; RUTF: Ready‐to‐Use Therapeutic Food; SAM: Severe Acute Malnutrition; WaST: Wasted and Stunted.

^a^ (WHZ < −3 and MUAC < 115 mm).

^b^ (WHZ < −3, MUAC < 115 mm, and edema).

^c^ (*n* = 586).

^d^ The health post in the child's kebele provides SAM treatment and had RUTF available at the time of the survey.

### 3.2 Characteristics of Caregivers and Fathers

The mean age of caregivers was 28.8 years (±7.0), while that of fathers was 36.0 years (±8.6). The majority of caregivers (92.9%) were married and lived with their spouse. Overall, 63.8% of caregivers reported having an income‐generating activity (Table 2).

**Table 2:** Caregiver and paternal characteristics of children 6–59 months with SAM in agrarian and pastoralist areas of Ethiopia, 2024 (*n* = 687).

Variables	Agrarian (*n* = 608)	Pastoralist (*n* = 79)	Total (*n* = 687)
**Caregiver/mother characteristics**			
Age of caregiver (years)	29.0 ± 7.1	27.3 ± 6.5	28.8 ± 7.0
Biological mother^a^	579 (95.4)	79 (100)	658 (95.9)
Married/living together with spouse	559 (92.1)	78 (98.7)	637 (92.9)
Caregiver currently pregnant	67 (11.0)	7 (8.9)	74 (10.8)
Total number of pregnancies in lifetime	4.2 ± 2.4	4.2 ± 2.5	4.2 ± 2.4
Total number of live‐born children	3.8 ± 2.3	3.9 ± 2.3	3.8 ± 2.3
Caregiver ever attended school	167 (27.5)	25 (31.6)	192 (28.0)
Caregiver has income generating activity	404 (66.6)	34 (43.0)	438 (63.8)
Probable depression^b^	157 (25.9)	15 (19.0)	172 (25.1)
Total EPDS score	9.2 ± 5.4	8.0 ± 5.1	9.1 ± 5.4
**Caregiver knowledge on:**			
SAM screening	0.1 ± 0.3	0.2 ± 0.3	0.1 ± 0.3
RUTF knowledge	0.2 ± 0.3	0.4 ± 0.3	0.2 ± 0.3
Breastfeeding knowledge	3.3 ± 1.4	3.6 ± 1.2	3.3 ± 1.4
Complementary feeding knowledge	2.8 ± 1.5	2.9 ± 1.3	2.8 ± 1.5
Child health/hygiene knowledge	1.4 ± 0.9	0.5 ± 0.6	1.3 ± 0.9
Total knowledge score 5 domains	1.6 ± 0.7	1.7 ± 0.7	1.6 ± 0.7
Total knowledge score without SAM and RUTF specific knowledge	1.3 ± 0.4	1.1 ± 0.3	1.2 ± 0.4
Caregiver perception of workload score	3.8 ± 2.5	4.3 ± 2.3	3.8 ± 2.5
Caregiver community involvement/support score	1.0 ± 1.6	2.1 ± 2.2	1.1 ± 1.8
Caregiver decision making power score	5.8 ± 3.2	6.4 ± 3.3	5.9 ± 3
Caregiver mobility score	6.5 ± 4.5	7.0 ± 4.1	6.6 ± 4.5
Caregivers' stigma perception	78 (12.9)	6 (7.6)	84 (12.2)
Caregiver had minimum dietary diversity (≥ 5 food groups)	124 (20.5)	15 (18.9)	139 (20.3)
Mother frequently absent from child	21 (3.5)	0 (0.0)	21 (3.1)
Characteristics of the father^c^			
Father has income generating activity	537 (95.5)	77 (97.4)	614 (95.8)
Age of father (years)	36.0 ± 8.4	35.4 ± 9.9	36.0 ± 8.6
Father ever attended school	161 (28.6)	36 (45.6)	197 (30.7)

*Note:* Data are presented as mean ± standard deviation (SD) for continuous variables and *n* (%) for categorical variables.

Abbreviations: EPDS: Edinburgh Postnatal Depression Scale; OTP: Outpatient Therapeutic Program; RUTF: Ready‐to‐Use Therapeutic Food; SAM: Severe Acute Malnutrition.

^a^ Caregiver is the biological mother of the child.

^b^ Edinburgh Postnatal Depression Scale score ≥ 13.

^c^ (*n* = 641).

#### 3.2.1 Treatment Coverage of SAM

The treatment coverage of SAM was 12.8% (95% CI: 9.0%–17.9%), with 12.3% in Kersa and 16.5% in Jeldessa. Although coverage was slightly higher among females (13.8%) compared to males (11.6%), this difference was not statistically significant. Additionally, coverage was higher among children aged 6–24 months (14.1%) compared to those older than 24 months (9.2%); however, this difference was also not statistically significant.

### 3.3 Perceived Barriers to SAM Treatment Seeking Among Caregivers, HEWs, and AFDs

**Figure 1:**
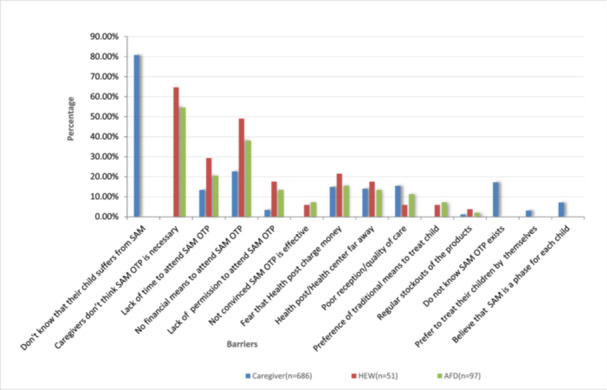
Caregivers, health extension workers and alliance for development leaders perceived barriers to SAM treatment seeking among 6–59 months children in agrarian and pastoralist areas of Ethiopia, 2024.

The most commonly reported barrier to seeking treatment for SAM from the caregivers' perspective was a lack of awareness that the child was undernourished, followed by financial constraints. HEWs, as well as AFD leaders, reported that the main barrier to seeking treatment for children with SAM was caregivers' lack of knowledge about the importance of SAM OTP services (Figure 1).

### 3.4 Determinants of SAM Treatment Coverage

Multivariable analysis identified several statistically significant factors associated with treatment coverage of SAM. After controlling for different confounders and the clustering effect, a higher household wealth index was positively associated with the likelihood of a child being under treatment. For each unit increase in the tertiles of the household wealth index, the likelihood of a child being under treatment increased by 2.9 (aPR = 2.9; 95% CI: 1.5–5.5). Children from households that used water treatment methods were more likely to be under treatment (aPR = 4.6; 95% CI: 1.7–11.9). The presence of SAM treatment services at first‐line health posts within a kebele was associated with a higher probability of a child being under treatment (aPR = 3.2; 95% CI: 1.1–9.9). Community‐based MUAC screening increased the likelihood of a child being under treatment. Children who were screened using MUAC in the community within the last 3 months prior to the survey were significantly more likely to be under treatment (aPR = 12.0; 95% CI: 5.4–26.8). Having a vaccination card was associated with the likelihood of being under treatment (aPR = 3.2; 95% CI: 1.1–9.1) (Table 3).

**Table 3:** Multivariable analysis of the predictors for treatment coverage of SAM among children 6–59 months in agrarian and pastoralist areas of Ethiopia, 2024 (*n* = 687).

Variables	Under SAM treatment		Bivariable analysis	Multivariable analysis
	No (*n* = 599)	Yes (*n* = 88)	cPR (95% CI)	aPR (95% CI)
Number of under five children	1.8 ± 0.6	1.7 ± 0.7	0.79 (0.6, 1.1)	—
Household wealth index			1.25 (0.9, 1.6)	2.9 (1.5, 5.5)**
Low	204 (34.1)	25 (28.4)		
Medium	199 (33.3)	30 (34.1)		
High	195 (32.6)	33 (37.5)		
HFIAS Score	6.9 ± 6.6	6.6 ± 6.1	0.98 (0.96, 0.99)*	—
Households used water treatment methods	33 (5.5)	16 (18.2)	2.08 (1.2, 3.7)*	4.6 (1.7, 11.9)**
Caregiver community involvement/support score	1.1 ± 1.7	1.6 ± 2.0	1.13 (1.02, 1.25)*	—
Caregiver Decision making power score	5.8 ± 3.2	6.9 ± 3.2	1.09 (1.01, 1.16)*	—
Caregiver perceived stigma	69 (11.5)	15 (17.2)	1.6 (1.02, 2.49)*	—
Caregiver had minimum dietary diversity (≥5 food groups)	115 (19.2)	24 (27.6)	1.42 (1.01, 1.99)*	—
Caregiver frequently absent from child	15 (2.5)	6 (6.9)	2.27 (1.06, 4.84)*	—
Father ever attended school	163 (29.2)	34 (41.5)	1.35 (0.89, 2.03)	—
Child age (months)	19.4 ± 13.3	16.6 ± 10.5	0.99 (0.97, 1.0)	—
Vaccination card available	98 (16.4)	26 (30.2)	1.61 (1.06, 2.45)*	3.2 (1.1, 9.1)*
Length/height‐for‐age Z‐score	−2.8 ± 1.4	–2.6 ± 1.2	1.07 (0.97, 1.17)	—
Child MUAC measured in the community past 3 months	20 (3.5)	8 (44.4)	12.3 (4.87, 31.2)***	12.0 (5.4, 26.8)***
Health post offers SAM OTP and has RUTF available^a^	164 (27.4)	33 (37.5)	1.48 (0.79, 2.73)	3.2 (1.1, 9.9)*
Jeldesa woreda (pastoralist)	66 (83.5)	13 (16.5)	0.95 (0.35, 2.6)	1.9 (0.57, 6.9)

*Note:* Data are presented as mean ± standard deviation (SD) for continuous variables and *n* (%) for categorical variables. Percentages were calculated from column totals.

Abbreviations: HFIAS: Household Food Insecurity Access Scale; MUAC: mid‐upper arm circumference; OTP: Outpatient Therapeutic Program; RUTF: ready‐to‐use therapeutic food; SAM: severe acute malnutrition.

* *p* < 0.05

** *p* < 0.01

*** *p* < 0.001.

^a^ The health post in the child's kebele provides SAM treatment and had RUTF available at the time of the survey.

## 4 Discussion

This study was aimed to assess the treatment coverage of SAM and identify key barriers and determinants among children aged 6–59 months in agro‐pastoral areas of Ethiopia. The findings revealed a period SAM treatment coverage of 12.8%, which falls below the 50% benchmark for rural settings recommended by the Sphere standard (Alliance for Child Protection in Humanitarian Action 2019). This low coverage in relatively stable areas suggests sub‐optimal implementation of Ethiopia's national SAM management guideline, resulting in a large proportion of children not accessing essential life‐saving treatment (FMOH, G. of E 2019). Furthermore, the Global Action Plan on Child Wasting calls for substantial scale‐up of treatment coverage by 2025 to support progress toward reducing wasting prevalence to below 5% (FAO, UNHCR, UNICEF, WFP, & W 2020). The observed coverage therefore indicates that the study area remains far from the level of scale‐up required to meet this global target.

The treatment coverage observed in this study aligns with findings from a multi‐country analysis conducted in four high‐burden African nations, where nine out of eleven regions reported treatment coverage rates below 20% (Heymsfield et al. 2025). These low coverage estimates underscore the widespread nature of the challenge in delivering effective SAM management across similar contexts. The latter estimates, however, were point coverage estimates not including children who were enrolled on SAM OTP while not being SAM at the time of the survey. The coverage rate identified in our study was lower than that reported in Nigeria (Isanaka et al. 2019). This disagreement may be attributed to methodological differences in case estimation. Our study used a broader SAM case definition (including the WHZ < –3 criterion), whereas the Nigeria study used a 2‐criteria definition (MUAC and edema).

Several factors were identified as being associated with SAM treatment coverage at the individual, household, community, and health system levels. The discussion was guided by the socio‐ecological model, which conceptualizes health service utilization as being influenced by interacting factors operating at multiple levels.

At the individual level, the barrier analysis identified limited caregiver awareness of child nutritional status as the most frequently reported barrier to seeking treatment for SAM. Many caregivers did not recognise that their child was malnourished, highlighting a critical gap in household‐level knowledge regarding the early identification of wasting. Similar patterns have been known in Ethiopia and other resource‐constrained settings, where poor understanding of SAM symptoms has been shown to delay care‐seeking behavior and contribute to low program coverage (Alelign et al. 2024; Manivannan et al. 2023; Puett and Guerrero 2015). The observed limited awareness also aligns with the very low community‐based screening coverage of wasting in the current study. More frequent screening by community groups like Alliance for Development groups or family members is likely to increase the awareness of child wasting and existing treatment services (Tamirat et al. 2025)(Majiwa et al. 2024).

At the household level, socioeconomic status was associated with SAM treatment coverage, with children from higher wealth households showing higher treatment seeking. This may be attributed to better access to financial resources, improved ability to afford transport costs, and greater exposure to health information, all of which may facilitate care‐seeking (Adedokun et al. 2017). This finding is supported by the barrier analysis, which identified financial constraints as the second most frequently cited challenge to seeking care. The finding is consistent with evidence from CMAM across sub‐Saharan Africa (Rogers et al. 2015). This might be due to the fact that, even if treatment itself is provided free of charge, caregivers frequently suffer indirect costs such as transportation, food, and opportunity costs of time spent away from household or livelihood responsibilities. These hidden costs have been shown to contribute to defaulting and reduced adherence to treatment protocols (Puett and Guerrero 2015).

Time constraints was another perceived barrier to treatment coverage. The finding is supported by the qualitative study done in Ethiopia, which revealed that high opportunity costs, specifically the time lost from essential agricultural and domestic activities, are important drivers of treatment coverage for SAM. While the Ethiopia CMAM Guidelines require weekly OTP visits until the child is discharged as cured (FMOH, G. of E 2019), this schedule can be difficult to follow in practice. For Agrarian households, the burden is particularly high during planting and harvest seasons, while for pastoralist households, fixed weekly appointments may not align with seasonal movement and livestock management. This barrier may be more significant in the present study, as data were collected from May to September, which is the main agricultural period in Ethiopia.

Children from households that used water treatment methods were also more likely to be under treatment. This association likely exceeds the biological benefits of safe water; rather, water treatment serves as a proxy for higher health literacy and stronger engagement with the health system. Caregivers who adopt these practices are often more likely to be reached by HEWs and are more integrated into preventative health services. Such factors may not only reduce the burden of infections that exacerbate malnutrition but also increase the likelihood that caregivers can recognize the signs of SAM and seek timely treatment (Li et al. 2018).

Having a vaccination card was associated with a higher likelihood of a child being on treatment. This might be due to the fact that having a vaccination card serves as a proxy indicator of households' engagement with the health system (Datta et al. 2016). Additionally, maintaining a vaccination card reflects a caregiver's health seeking behavior and trust in formal services, both of which contribute to timely identification and treatment of malnourished children. On the top of this, the association may also reflect a cluster‐level effect of health posts, where better‐performing health posts are more likely to provide both curative services and preventive services, leading to higher uptake of immunization and improved identification and referral of SAM cases within their catchment areas.

Besides the factors on household and caregiver level, our analysis also identified two important factors at the treatment service supply side. First, at the community level, there was an association between a SAM child being screened in the community in the last 3 months preceding the survey and the likelihood of a being under treatment. Which may be due to the fact that active community‐based screening as opposed to passive screening of cases during health facility consultations is likely to detect SAM earlier (J. Bliss et al. 2018) and may tackle barriers such as geographic and financial barriers, obtaining permission from the father or other family members, and social stigma to attend health facilities (Puett and Guerrero 2015) (Tanou and Kamiya 2019). Despite this, our findings show that only 4.8% of children with SAM had been screened in the community in the last 3 months prior to the survey. This represents gap in adherence to the Ethiopia CMAM Guidelines, which mandate active case‐finding as a core strategy (FMOH, G. of E 2019). To address this gap, it is essential to strengthen active case‐finding by HEWs and the AFDs. Additionally, adopting the Family MUAC approach whereby caregivers are trained to monitor their own children's nutrition status represents a high‐impact, low‐cost complement to existing outreach strategies (GOAL 2019) (Alé et al. 2016). Evidence has shown that Family MUAC can significantly increase early detection and treatment seeking without overextending the formal health workforce (Tamirat et al. 2025). Second, at the health system level, we found that children residing in kebeles where nearby first‐line health posts offer SAM treatment with RUTF supplements available at the time of the survey, were more likely to be under treatment. While Ethiopian national policy orders the decentralization of SAM treatment to the community level via health posts our findings reveal a profound gap between policy and implementation (FMOH, G. of E 2019). Although health post were functioning across all study kebeles, only 28.7% were providing SAM treatment services with available RUTF at the time of the survey. Children residing in these “service‐ready” kebeles were significantly more likely to be under treatment. This may be due to the fact that, in case a nearby health post does not offer SAM treatment service caregivers need to take their children to more remote areas which leads to logistical and possibly financial challenges to attend the weekly SAM treatment visits (Rogers et al. 2015).

In this study, Jeldessa (pastoralist) woreda showed a higher proportion of health posts offering SAM treatment services (60.8%) and a slightly higher treatment coverage (16.5%) compared to Kersa. However, the multivariable analysis did not show a statistically significant association between woreda and treatment coverage. This lack of significance is likely due to the small sample size in Jeldessa, which resulted in wider confidence intervals and reduced statistical power to detect a woreda‐level effect. Despite the lack of statistical significance, the descriptive gap suggests that better‐equipped health systems in pastoralist areas may improve access, but further studies with larger, balanced samples are needed to confirm these differences.

The key strength of this study is the estimation of SAM treatment coverage across two woredas by enrolling the entire population of children identified with SAM or receiving SAM treatment services. Although this approach is resource‐intensive, it provides a more valid estimate of coverage compared to semi‐quantitative methods (Isanaka et al. 2019). However, several limitations of this study should be acknowledged when interpreting the study findings. First, the cross‐sectional design of the study does allow for direct causal inferences between the identified associated factors and SAM treatment coverage; therefore, the associations observed should be interpreted as exploratory. Additionally, the purposive selection of study sites based on SAM prevalence and accessibility may limit the generalizability of the findings to other settings in Ethiopia. Furthermore, the small number of children with SAM under treatment (12.8% of the sample) resulted in limited statistical power for certain variables in the multivariable regression model, as reflected in the wide confidence intervals. While these point estimates are directionally informative, they should be interpreted with caution. There is also a possibility of residual confounding. For instance, variables such as water treatment use and possession of a vaccination card may serve as proxies for unmeasured factors rather than direct association of treatment coverage. A potential for ecological fallacy exists regarding the health post variable, which was assessed at the kebele level. In addition, as data collection occurred between May and September 2024, the findings reflect the main rainy and lean seasons; consequently, SAM prevalence and health‐seeking behaviors may differ from other periods of the year. We used a conservative operational definition to estimate treatment coverage based on three criteria: caregiver acknowledgment of OTP enrollment, consumption of RUTF within the last 3 days, and visual confirmation of at least one full RUTF sachet or more than one empty sachet. However, the second criterion (consumption of RUTF within the last 3 days) and the third criterion (visual confirmation of RUTF sachets) may have the potential to underestimate treatment coverage, as children who were actively enrolled in OTP might not have fulfilled these criteria during the survey visit due to different factors, such as RUTF stockout or missing the last OTP visit despite still being on active treatment. Yet, in this study, only two children were excluded after applying the second and third criteria, suggesting minimal effect on the overall coverage estimate. Also, R‐out data (recovering cases outside the program) were not available in this study. Thus the single coverage estimator was not computed as it requires complete information on both covered and uncovered recovering cases. This could have affected the coverage estimation.

As conclusion, this study highlights a low SAM treatment coverage in the study area. The gap is largely attributed to individual and household‐level barriers, particularly limited caregiver awareness of child nutritional status and the burden of indirect costs associated with seeking care. In contrast, treatment coverage was significantly higher among children from households with higher socioeconomic status and among those who were engaged in preventive health services such as vaccination consultations. On the service supply‐side, a strong association was observed between SAM treatment status and the presence of community‐based screening, as well as accessibility of treatment sites for SAM at nearby health posts. These findings emphasize the essential role of proactive and accessible healthcare in reducing the burden of SAM and the associated child mortality. To address the identified gaps, it is recommended to implement a tiered programmatic framework that integrates both supply and demand‐side determinants in alignment with national guidelines. At the supply level, the Federal Ministry of Health and its partners could further strengthen the consistent delivery of OTP services by ensuring that every kebele health post maintains a consistent RUTF supply, which would make decentralized care more functionally available to all affected children. This effort could be supported at the community level by encouraging Family MUAC screening alongside monitoring of active case‐finding by HEWs and community volunteers. At the household and individual level, addressing indirect costs and creating awareness to caregivers through targeted health education would be essential toward overcoming socioeconomic barriers and ensuring the timely, equitable delivery of SAM treatment.

## Author Contributions

Conceptualization: Lieven Huybregts, Tefera Belachew, Bayise Biru, Alemayehu Haddis. Supervision: Tefera Belachew, Lieven Huybregts, Bayise Biru, Mohammed Areb, Dessalegn Tamiru, Alemayehu Haddis. Data curation: Bayise Biru, Lieven Huybregts, Mariama Touré, Tefera Belachew, Mohammed Areb, Dessalegn Tamiru, Talla Fall. Formal analysis: Bayise Biru, Lieven Huybregts, Mariama Touré, Tefera Belachew, Talla Fall writing – original draft of the Manuscript, Bayise Biru. Writing – Review and editing: Lieven Huybregts, Tefera Belachew, Dessalegn Tamiru, Talla Fall, Mariama Touré, Alemayehu Haddis. All Authors read and approved the final manuscript.

## Conflicts of Interest

The authors declare no conflicts of interest.

## Supporting information


**Table S1:** Technical Error of measurement standardization result.

**Table S2:** Assets used in the PCA to generate wealth index, and the factor loading.

## Data Availability

The data that support the findings of this study are available on reasonable request from the corresponding author. The data that support the findings of this study are available on request from the corresponding author. The data are not publicly available due to privacy or ethical restrictions.
