# Supplementary material for: Barriers and Factors Associated With Treatment Coverage of Severe Acute Malnutrition Among Children 6–59 Months in Agrarian and Pastoralist Areas of Ethiopia
Source: Matern Child Nutr. 2026 Jul 1;22(3):e70219. doi: 10.1111/mcn.70219 (PMC13320365; doi:10.1111/mcn.70219)
Supplement: Supplementary file 2 — Table S2: Assets used in the PCA to generate wealth index, and the factor loading. [file MCN-22-e70219-s001.docx]

Table s2: Assets used in the PCA to generate wealth index, and the factor loading

| Assets/wealth | Factor loading on component 1 |
| --- | --- |
| Functioning Radio | 0.32 |
| Watch(Hand/wall | 0.35 |
| plough | 0.32 |
| Grass matress | 0.28 |
| Chair/stool | 0.31 |
| Solar panel | 0.28 |
| Having Bank account | 0.29 |
| Being a member of CBHI | 0.20 |
| Any cows/Bull | 0.17 |
| Any Horse/Donkey/Mules | 0.15 |
| Chicken/poultry | 0.21 |
| Sheep/Goats | 0.13 |
| Beehive | 0.11 |
| Own agricultural land | 0.24 |
| Number of sleeping rooms | 0.28 |

Note: The first principal component explained 22.4% of the total variance.
